# Is “Football for All” Safe for All? Cross-Sectional Study of Disparities as Determinants of 1-Year Injury Prevalence in Youth Football Programs

**DOI:** 10.1371/journal.pone.0043795

**Published:** 2012-08-22

**Authors:** Örjan Dahlström, Stefan Backe, Joakim Ekberg, Staffan Janson, Toomas Timpka

**Affiliations:** 1 Department of Medical and Health Sciences, Linköping University, Linköping, Sweden; 2 Linnaeus Centre HEAD, Swedish Institute for Disability Research, Linköping, Sweden; 3 Department of Behavioral Sciences and Learning, Linköping University, Linköping, Sweden; 4 Division of Public Health Sciences, Karlstad University, Karlstad, Sweden; 5 Department of Public Health, School of Life Sciences, Skövde University, Skövde, Sweden; University of Granada, Spain

## Abstract

**Background:**

Football (soccer) is endorsed as a health-promoting physical activity worldwide. When football programs are introduced as part of general health promotion programs, equal access and limitation of pre-participation disparities with regard to injury risk are important. The aim of this study was to explore if disparity with regard to parents’ educational level, player body mass index (BMI), and self-reported health are determinants of football injury in community-based football programs, separately or in interaction with age or gender.

**Methodology/Principal Findings:**

Four community football clubs with 1230 youth players agreed to participate in the cross-sectional study during the 2006 season. The study constructs (parents’ educational level, player BMI, and self-reported health) were operationalized into questionnaire items. The 1-year prevalence of football injury was defined as the primary outcome measure. Data were collected via a postal survey and analyzed using a series of hierarchical statistical computations investigating associations with the primary outcome measure and interactions between the study variables. The survey was returned by 827 (67.2%) youth players. The 1-year injury prevalence increased with age. For youths with parents with higher formal education, boys reported more injuries and girls reported fewer injuries than expected; for youths with lower educated parents there was a tendency towards the opposite pattern. Youths reporting injuries had higher standardized BMI compared with youths not reporting injuries. Children not reporting full health were slightly overrepresented among those reporting injuries and underrepresented for those reporting no injury.

**Conclusion:**

Pre-participation disparities in terms of parents’ educational level, through interaction with gender, BMI, and self-reported general health are associated with increased injury risk in community-based youth football. When introduced as a general health promotion, football associations should adjust community-based youth programs to accommodate children and adolescents with increased pre-participation injury risk.

## Introduction

Football (soccer) is presently endorsed as a health-promoting physical activity in communities worldwide. [1], [2] The game is already played regularly by more than 265 million players, most of them children and youth. [3] In Sweden, it is estimated that almost 1 million people (11.2% of the total population) play football. [3] When community-based football programs are introduced in a population health context, elimination of pre-participation disparities is important because equity and sustainability are essential constituents of health promotion. [4] Previous studies have shown that football injury rates are low among younger children [5], [6] but increase during late adolescence to up to 37 injuries per 1000 hours of play. [7] Other factors known from general epidemiological studies to be associated with risk of injury and disease have more seldom been studied in the youth sports setting. However, children with high body mass index (BMI, calculated as weight in kilograms divided by the square of height in meters) participating in sports have been reported at increased injury risk. [8], [9] Existing research has also indicated that girls involved in sports [10] and children from less resourceful households [11], [12] are at increased risk for injury. It has likewise been suggested that players from nonacademic families are brought up to play through injury and pain, as a continuation of a working-class cultural tradition. [13] Though, it has not been investigated what importance such pre-participation factors, including general health status [14], have for injury risk in community football programs, and whether the effect of any one of these is enforced by another or by age or gender.

This study sets out to explore if parents’ educational level, player body BMI, and self-reported health are determinants of injury in community-based football programs, separately or in interaction with age or gender. Knowledge of pre-participation disparities can be used by football associations and clubs when planning community-based programs for broad youth populations.

## Materials and Methods

A cross-sectional study design was used accounting for retrospectively reported player-level data from one study season (Text S1). Data were collected via a postal questionnaire. The 1-year prevalence of football injury was used as the primary outcome measure in the analyses. The results were reported according to the STROBE statement for epidemiologic studies. [15] The study design was approved by the Regional Research Ethics Committee at Karlstad University (Dnr. C2006/474). All youth players belonging to the clubs at the start of the study season and their parents (for players under 15 years of age) received information in writing about the study and were asked to give their written consent to participation. A consent form signed by a guardian on the behalf of the minors/participants involved in the study was returned with each survey document. Minors older than 15 years of age were only required to sign the form by themselves. The age of consent was determined by the upper age limit for pediatric care in Sweden. The participants were anonymized by replacing personal identification data with a code in the database. This procedure was approved by the Regional Research Ethics Committee.

### Study Population

Four community football clubs, representing both large cities and smaller communities as well as professional and amateur organizations (paid staff vs. volunteers) were invited to participate in the study. Each club accounted for about 300 youth players. Following board decisions, all clubs agreed to take part in the study during the 2006 season (reaching between December 2005 and November 2006). Two clubs (Club A and Club B) were the largest clubs for youth football within their respective urban communities (municipalities with 50,000–200,000 inhabitants and more than 70% urban areas). These 2 clubs also cooperated with elite clubs in the Swedish professional leagues. The 2 other clubs (Club C and Club D) were situated in suburbs of a large city (population 1,500,000). These clubs had no formal cooperation with professional clubs and their first teams played in regional amateur leagues.

In Sweden, youth football competitions are organized in birth-year-specific leagues. Accordingly, the study clubs mainly organized birth-year-specific teams for both boys and girls, but no club had a birth-year-specific team for each age and gender group. Three of the clubs had merged girls from 2 adjacent age groups to form teams, and the clubs shared only 3 teams at the junior level (17–18 years) between them, 2 boys’ teams and 1 girls’ team. The final eligible study population consisted of 1,230 youth players.

### Definitions

In this study, football injury was defined as “an event occurring during a football match or training session that caused the player to miss at least one scheduled match or team training session or required medical attention”. As in the New Zealand Soccer (NZS) community football study [7], collection of data on “any physical complaint irrespective of the need for medical attention or time loss” as prescribed at the highest level of the Fédération Internationale de Football Association (FIFA) definition [16] was found not applicable to children. The operationalization of the remaining study constructs into questionnaire items is displayed in Table 1.

**Table 1:** Operationalization of study constructs.

Construct	Measure	Comment
Gender	Female, male	Questionnaire item
Age	Full years	Questionnaire item
Age group	8–10, 11–12, 13–14, 15–18 years	Constructed variable. Used in some analyses
Injury	Ordinal scale (1, 2–3 or ≥4 injuries)	Questionnaire item. The self-reported number of injuries during training and matches (during the study year). The answer was given on an ordinal scale (0 injuries, 1 injury,2–3 injuries or ≥4 injuries)
Parents’ educational level	High, low	Constructed variable. The formal education of the players’ parents was asked forin the questionnaire. The highest formal education of the highest educatedparent was used to define the level: High, at least one parent with a universitydegree; low, otherwise
Body mass index	z-Height, z-BMI	Constructed variable. Self-reported height and weight were asked for in the questionnaire. Height and BMI were transformed into gender- and age-definedz-scores, i.e., number of gender- and age-specific standard deviations an individual differs from his/her gender- and age-specific mean
Self-reported health	Full health, low health	Constructed variable. Self-reported health was initially reported on a three-item scale. Full health, very healthy; low health, quite healthy or not very healthy

### Data Collection

Data were collected 1 month after the end of the studyseason. All youth players belonging to the clubs at the start of the study season and their parents (for players under 15 years of age) received information in writing about the study and were asked to give their written consent to participation. The consent could be withdrawn at any time during the study without specifying the reason. A postal survey asking for data regarding the past season was then sent to those consenting to participation. The questionnaire contained predominantly closed items (tick the box format). The survey was resent once to non-respondents.

### Statistical Analyses

The statistical computations consisted of a series of hierarchical analyses aiming to answer the research questions (Q1–Q4) corresponding to the study aims (Table 2). All statistical analyses were performed using IBM SPSS 19.0.

**Table 2:** Research questions and corresponding variables.

Research Question: Are injuries evenly distributed over…	Variables introduced into analyses
Q1. … gender and age?	Injuries, gender, age group
Q2. … gender, age and *parents’ educational level*?	Injuries, gender, age group, *parents’ educational level*
Q3…. gender, age and *body mass index*?	Injuries, gender, age group, *player body mass index (z-BMI, z-Height, z-Weight)*
Q4. … gender, age and *self-reported health*?	Injuries, gender, age group, *self-reported health*

For question 1, all interactions were of interest starting with the highest-order statistically significant interaction. For questions 2–5, the highest-order significant interaction was of interest, but only if it contained the injuries variable and the question-unique variable (in *italics*).

In the first step (answering Q1), interaction effects associated with 1-year injury prevalence were investigated for the gender and age groups at hand. Initially, differences in 1-year injury prevalence between girls and boys were analyzed by independently investigating the number of boys and girls exposed to injury risk, and the number of individuals from each gender category reporting and not reporting injuries using 95% confidence intervals.

In the second step of the analyses (comprising Q2 and Q4), the approach was extended to more variables, always testing a highest-order interaction (in our case a 4-way interaction) and, if not significant, excluding it and testing the next highest-order interactions (in our case 3-way interactions), and so on (in our case 2-way interactions). The test used to compare the observed with the expected frequencies was a log-linear analysis in this case. Each subgroup was examined with regard to the standardized residuals, that is, standardized measures for how much the specific subgroup differed from what would be expected. In general, standardized residuals less than –2 indicate unexpectedly small frequencies and standardized residuals larger than 2 point toward unexpectedly high frequencies. In the case of significance but no standardized residuals of that size, the largest standardized residuals are reported. Cramer’s V was used as a measure of effect size and for two-way interactions between dichotomous variables, the Rothman synergy index [17], [18] was reported to show the excess risk from exposure to both exposures when there is interaction relative to the risk from exposure without interaction [19], [20], that is, when there is no interaction, the synergy index is close to 1. Notably, no expected frequency should be less than 1 and not more than 20% of the expected frequencies should be less than 5, therefore one cannot easily divide, for example, age into too fine a structure (i.e., that is why age groups are used instead of full years).

The BMI of our sample was also compared with reference values. [21] The relation between BMI and injuries (Q3) was then investigated by comparing the gender- and age-defined z-scores of youths reporting injuries and those who did not, using independent samples t tests.

## Results

### Participants

The survey was returned by 827 (67.2%) of the youth players. Sixty (4.8%) players actively chose not to participate in the study. In total, 767 (62.4%) youth players provided complete data sets (Table S1). There was no gender difference in the mean exposure to football practice and games, which ranged from about 4 hours per week among the youngest players to more than 7 hours among the players aged 15–18 years (Table 3). About 1 in 3 children in the younger age groups had parents with low education, decreasing to 1 in 4 of the older children. In contrast, less than 1 in 10 of the youngest children reported low self-reported health, compared with almost every third girl and every fifth boy in the oldest group.

**Table 3:** Training hours, parents’ educational level, and self-reported health (95% Confidence Intervals) of the study participants (*n* = 767) displayed by gender and age.

	Gender	Age 8–10 years		Age 11–12 years		Age 13–14 years		Age 15–18 years	
		Mean	(CI)	Mean	(CI)	Mean	(CI)	Mean	(CI)
Training hours per week, mean (CI)^a^	Girls	3.4	(2.2–4.5)	4.6	(3.4–5.8)	6	(4.8–7.2)	4.8	(2.9–6.7)
	Boys	3.7	(2.7–4.7)	5.8	(4.7–6.8)	6.4	(5.3–7.6)	7.6	(6.2–9.1)
Parents with lower education, % (CI)	Girls	29	(19–40)	34	(24–44)	24	(15–33)	24	(10–37)
	Boys	37	(29–45)	28	(21–36)	33	(25–42)	24	(14–34)
Low self-reported health, % (CI)	Girls	4	(0–8)	16	(8–23)	13	(5–20)	28	(14–41)
	Boys	9	(4–14)	14	(8–19)	13	(7–19)	19	(10–28)

a Individuals’ reported mean training hours/week during the outdoor season.

Girls had on average higher BMI than reference groups (t(9) = 4.77, P = .001, r = .85) (Table S2). Only at age 12 years did the BMI of the girls fall short of the reference values. Boys had on average 0.29 standard deviations higher BMI than reference groups (t(9) = 5.52, P<.001, r = .88). They had higher BMI than the reference groups at all ages.

### One-Year Injury Prevalence and Interactions between Injuries, Gender and Age (Q1)

The general 1-year injury prevalence (having sustained ≥1 injuries during the study season) was higher among boys than among girls for all age groups (Table 4). The lowest injury prevalence was recorded for the players aged 8–10 years. For both genders, the highest 1-year injury prevalence was observed in the 15–18 years age group (girls 74%, boys 77%). Among the players aged 15–18 years, 27% of the boys and 21% of the girls sustained 4 or more injuries.

**Table 4:** One-year injury prevalence in percent (95% Confidence Intervals) displayed by gender and age.

Injuries	Gender	Age 8–10 years		Age 11–12 years		Age 13–14 years		Age 15–18 years	
		Mean	(CI)	Mean	(CI)	Mean	(CI)	Mean	(CI)
≥1 injuries	Girls	26	(16–36)	59	(49–69)	66	(56–77)	74	(60–88)
	Boys	27	(20–35)	61	(53–69)	75	(67–83)	77	(68–86)
≥2 injuries	Girls	19	(10–28)	38	(28–48)	45	(34–57)	50	(34–66)
	Boys	15	(9–21)	38	(30–46)	56	(47–65)	59	(48–70)
≥4 injuries	Girls	4	(0–9)	18	(10–26)	16	(7–24)	21	(8–34)
	Boys	7	(2–11)	12	(7–17)	20	(12–27)	27	(17–37)
Total answers	Girls	73		90		77		38	
	Boys	136		141		117		78	

### Interactions between Injuries and Parents’ Educational Level (Q2)

**Figure 1:**
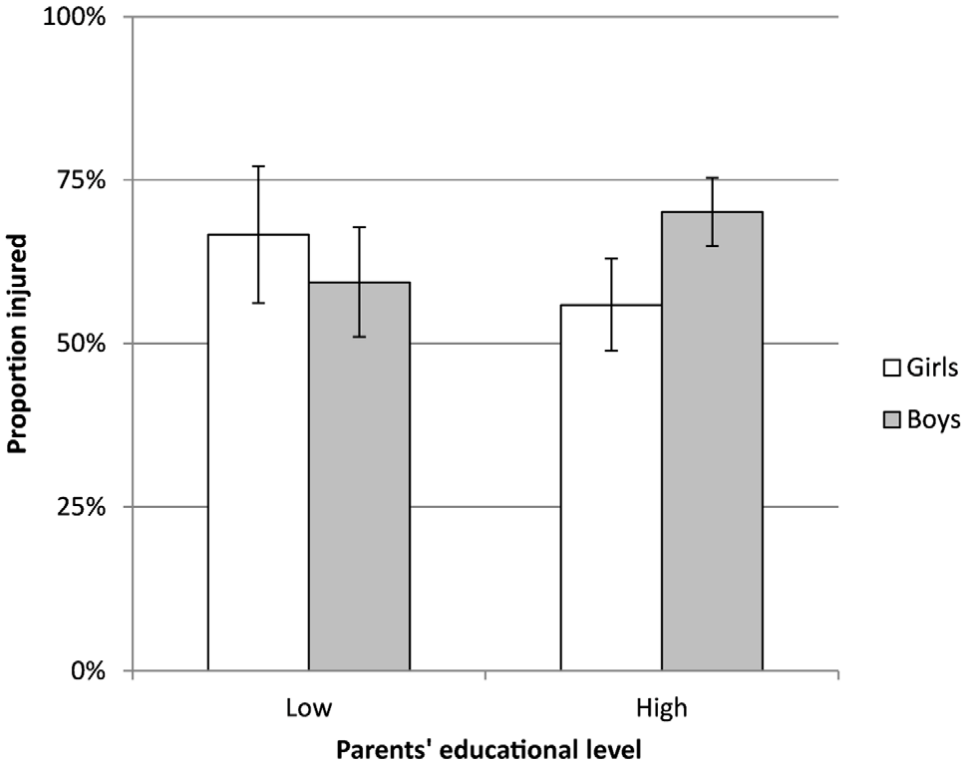
Relative number of injuries. Relative number of injuries (with 95% confidence intervals) for girls and boys with parents at higher and lower educational levels.

There was no significant 4-way interaction between parents’ educational level, injury, age, and gender (χ^2^(3, N = 677) = 0.31, P = .96). After backward elimination of nonsignificant higher-order effects, a significant 3-way interaction between parents’ educational level, injury, and gender remained (χ^2^(1, N = 677) = 6.71, P = .01) (Figure 1). This indicates that the association between injury and gender differed between players having parents at low and high educational level, respectively. For youths with parents at high educational level, boys reported injuries to a higher degree and girls reported injuries to a lower degree than expected (χ^2^(1, N = 474) = 9.99, P = .002, Cramer’s V = .15, Rothman’s synergy index = 4.62). For youths with parents at the lower educational level, there was no significant difference but a small tendency toward the opposite pattern (χ^2^(1, N = 203) = 1.07, P = .30, Rothmans’s synergy index = 0.98) (Figure 1).

To rule out club membership as a confounding factor, interactions between club, parents’ educational level, and gender were examined. The log-linear analysis showed no significant 3-way interaction (χ^2^(3, N = 751) = 5.64, P = .13), but two 2-way interactions; club × parents’ educational level and club × gender. It was found that the proportion of individuals with a specific educational level of the parents differed between the clubs (χ^2^(3, N = 751) = 11.34, P = .01, Cramer’s V = .12). Parents at the lower educational level were underrepresented in Club C (standardized residual = −2.1) and slightly overrepresented in Club D (standardized residual = 1.8). There were also gender differences between the clubs (χ^2^(3, N = 751) = 27.64, P<.001, Cramer’s V = .19). However, the gender difference differed from the difference in parents’ educational level; here the differences were explained by discrepancies in Club A and Club B. Boys were overrepresented in Club A (standardized residual = 2.8) and slightly underrepresented in Club B (standardized residual = −1.2), whereas girls were underrepresented in Club A (standardized residual = −3.6) and slightly overrepresented in Club B (standardized residual = 1.6). This means that the interaction between parents’ educational level and gender cannot be explained by club-specific factors.

### Interactions between Injuries and BMI (Q3)

Based on gender- and age-standardized values (based on the estimated population means and standard deviations), youths reporting injuries had on average 0.19 standard deviations higher BMI compared with youths not reporting injuries (t(577) = 2.66, P = .008, r = 11, corrected for unequal variances between groups). Those reporting injuries were also on average 0.21 standard deviations heavier than those who did not report injuries (t(688) = 2.74, P = .006, r = .10). In addition, those reporting injuries were on average 0.15 standard deviations taller than those who did not report injuries (t(688) = 1.91, P = .06).

### Interactions between Injuries and Self-reported Health (Q4)

There was no significant 4-way interaction between self-reported health, injuries, gender and age group (χ^2^(3, N = 688) = 4.23, P = .24). Backward elimination of nonsignificant higher-order effects resulted in 2 significant 2-way interactions. The first 2-way interaction was between self-reported health and injuries (χ^2^(1, N = 688) = 8.80, P = .003, Cramer’s V = .11, Rothman’s synergy index = 0.24). Children not reporting full health were slightly overrepresented among those reporting injuries (standardized residual = 1.7) and underrepresented for those reporting no injury (standardized residual = −2.2). The second 2-way interaction was between self-reported health and age group (χ^2^(1, N = 764) = 14.97, P = .002, Cramer’s V = .14). Children not reporting full health were underrepresented in the group aged 8–10 years (standardized residual = −2.5) and overrepresented in the group aged 15–18 years (standardized residual = 2.5).

## Discussion

We set out to explore if pre-participation disparity with regard to parents’ educational level, player BMI, and self-reported health were determinants of football injury in community-based football programs, separately or in interaction with age or gender. Parents’ educational level alone, as measured by their formal education, was not found to be associated with injury risk. However, in children with parents having received a higher education, boys reported injuries more often than girls; there were slight indications the opposite was the case for children having parents with lower education. This interaction pattern could not be explained by club-level factors. “Doing gender” has been used to describe when gender is constituted through social interaction [22], that is, differences between girls and boys that are not biological are created in day-to-day social contexts. Organized sports have been cited as a particular example of institutionalized expression of manliness. [23, p.322] Qualities such as endurance, strength, and competitive spirit are aimed for by all parties involved, participants as well as coaches, managers, and spectators. One hypothetical explanation for the interaction pattern observed in this study is that causative factors for youth football injury risk are to be found in the players’ family environment. Parents with academic education may to a higher degree have conformed to the prevailing expressions of gender in sports, which were mediated to their daughters and sons through family interactions and eventually transformed into behaviors on the football field. According to this hypothesis, boys from academic families may thus have been encouraged to be more competitive and thereby became more prone to expose themselves to injury risks than their peers from nonacademic families. This direction of the interaction suggests that the causes of the variations in the injury rates between girls and boys with parents at low and high educational levels can be sought in demands and expectations, rather than in depravation of material resources, for example, inadequate equipment.

We observed that the youth football players in our study displayed tendencies for higher BMI values than the reference population of the same age. A likely interpretation of this observation is that this difference not was due to fat mass, but that football players had a larger lean body mass than children of the same age. We also found a positive association between high age-adjusted player BMI and increased injury risk, that is, individuals with larger body mass relative to their player peers displayed an increased injury risk. In a review of 13 studies [24], 11 reported evidence that overweight children are at increased risk of sustaining sports injuries. Mechanisms proposed to mediate this risk include poor postural control (leading to problems with balance and coordination), poor physical fitness (associated with muscle fatigue and subsequent injury), and low pre-participation physical activity levels (associated with impaired neuromuscular and motor learning). It has been reported that participation in football programs is as efficacious in improving the physical capacity, health-related fitness parameters, and self-esteem of overweight children as a standard exercise program. [25] We interpret that the slightly increased injury risk is not a reason to discourage overweight children participating in football programs. Instead, we suggest that Carter and Micheli’s general recommendations [26] for training of the child athlete should also be applied to youth football, that is, youth football programs must be individual-, sport- and context-specific, taking into account factors such as a child’s age, BMI, developmental level, and skill set. Modifiable risk factors, including poor postural control and physical fitness, should be identified and addressed to ensure that overweight children can participate in football activities as safely as possible.

We found 10–20% of the adolescent players reported less than optimal health and that low self-reported health was associated with increased injury risk. In our study population, asthma and allergies were the most common self-reported medical problems (unpublished data). Pre-participation cardiovascular screening of young athletes is formalized in some countries and a legally regulated activity. [27] How to consider pre-participation screening is a much debated topic [28] and the observations in our study actualize to consider pre-participation by also taking other prerequisites such as socio-economic factors into consideration. However, it should be kept in mind that a pre-participation examination in youth football should not be restricted to identifying individuals at risk for sudden cardiac death; the goal of these examinations should be to expose any medical problems before participation to be able to prevent injuries and exaggerated health problems. Our results point out that in community-based football programs, there is a need to create awareness among coaches and club officials of common health issues among players. These issues may interfere with the players’ fitness or coordination and increase their injury risk. Information from a focused pre-participation medical history can be used to prevent the occurrence of an injury or illness. In an American study involving 239 young athletes [29], key issues detected using such a history form included a probable history of asthma, a family history of cardiac death before the age of 50 years, a history of previous joint injury, and the use of medications for chronic medical problems. Of these most frequently encountered problems, only musculoskeletal injury would be likely to be detected without a history form. We also echo that modifiable risk factors, including poor physical fitness and medications, should be identified and addressed to ensure that children with medical problems can participate in football activities as safely as possible. It is the ultimate responsibility of involved adults (coaches, parents, and club officials) to ensure the safety of each player.

There are several potential sources of bias that need to be taken into consideration when interpreting the results of this study. Our sample of youth players from clubs in urban and suburban areas was chosen to be representative for Sweden with regard to club policies and sociodemographic settings. There may be other specific contexts (e.g., football academies and small clubs in rural areas) where the results do not apply. However, we consider the external validity to be satisfactory for most youth football settings in Scandinavia; the generalization to other contexts must be made with care. There are other non-controlled structural factors that could have had influence on the results (coaches’ characteristics, facilities, time of exposure, etc.). This study addressed only a limited set of pre-participation factors with possible association to injury risk. Further studies of factors associated with the local organization of the youth football programs are warranted. Additionally, it must be acknowledged that computing of BMI from self-reported weight and height data is inferior to using data from standardized measurements. However, validation studies of self-reported BMI have reported high correlations between measured and self-reported BMI [30], [31] for use in epidemiological studies. We estimated the 1-year injury prevalence from retrospectively collected data based on self-reports. This approach is inferior to prospective designs used in, for instance, the NZS surveillance system for youth football [7] for recording data from exposure and injury events; for example, injury mechanism, severity (time loss), and classification. However, in this study of pre-participation disparities, there was no ambition to record biomedical and contextual data from injury events. Despite the design differences, there are similarities between our results and those reported from previous studies. Our findings that about 40% of female adolescent players and about 60% of male adolescent players were injured at least once during a season are consistent with those reported for some previous studies [7], [32], although other studies report that more than 2 players out of 3 were injured in the course of a season. [33], [34] Nonetheless, the 1-year injury prevalence is dependent on person-time exposure to football training and practice. The players in our study population recruited from community-based programs may have played less frequently than elite-level players participating in other studies. In contrast to some previous studies in youth soccer where no gender difference was observed [35], [7], we found that the 1-year injury prevalence was higher among boys. In accordance with previous studies from Canada [35] and Sweden [6], we found that the injury prevalence increased with age. Because the data only were collected during only one season, it is not possible to tell whether this pattern reflects biological factors, such as end-pubertal growth, or that some older players had left the community clubs aiming for a professional career or as a consequence of injury. A consensus statement on injury definitions and data collection procedures was published after this study began. [16] Our definition of injury was not as broad as that prescribed, in that our threshold for inclusion was “missing at least one scheduled match or team training or receiving medical attention,” whereas the consensus statement prescribed “any physical complaint … irrespective of the need for medical attention or time loss.” The reporting of prevalence rates in this study is still consistent with the consensus statement, although our classification of injury is not in the form prescribed. However, we recognize that in our study in the youth football setting, it is likely that there were age differences regarding what symptoms were acted upon as injuries, that is, what damage to bodily structures and functions, with adjoining pain, ended up being recorded as a time-loss injury. This issue warrants investigation in future studies. Finally, the epidemiological term interaction denotes that the effect of one risk factor is modified by another risk factor. Interaction in this study is defined as a deviation from additives of the absolute associations between risk factors, meaning that the combined association of the exposures is more or less than the sum of the separate associations. [36] The additive scale is commonly used when numbers of events (e.g., injuries) are counted and conclusions in this study are based on such additive interactions with potential implications for community-based football programs.

Our results demonstrate that parents’ educational level, through interaction with gender, BMI, and self-reported general health, are associated with pre-participation injury risk in youth football programs. Recognizing that football is introduced as a means to promote physical activity, our results can be used to inform development of safety policies for broad youth football programs taking into regard how children differ in their pre-requisites for safe participation.

## Supporting Information

Table S1 Invited study population and study participants.
(DOC)

Table S2 BMI values for the sample and a Swedish reference population.
(DOC)

Text S1 Details of research program.
(DOC)
