# Supplementary material for: Is “Football for All” Safe for All? Cross-Sectional Study of Disparities as Determinants of 1-Year Injury Prevalence in Youth Football Programs
Source: PLoS One. 2012 Aug 22;7(8):e43795. doi: 10.1371/journal.pone.0043795 (PMC3425492; doi:10.1371/journal.pone.0043795)
Supplement: Table S1 — Invited study population and study participants. (DOC) [file pone.0043795.s001.doc]

# Supporting information Table S1.

Study participants: numbers of participants/eligible subjects (%) displayed by age and club.

|  | Club A | | Club B | | Club C | | Club D | |
| --- | --- | --- | --- | --- | --- | --- | --- | --- |
| Age | Boys (%) | Girls (%) | Boys (%) | Girls (%) | Boys (%) | Girls (%) | Boys (%) | Girls (%) |
| 8 | 8/20 (40.0) | – | 21/34 (61.8) | 15/18 (83.3) | 11/20 (55.0) | 5/14 (35.7) | 8/14 (57.1) | 10/17 (58.8) |
| 9 | 15/25 (60.0) | 7/15 (46.7) | 12/18 (66.7) | 11/19 (57,9) | 14/18 (77.8) | 8/13 (61.5) | 5/8 (62.5) | 6/14 (42.8) |
| 10 | 9/21 (42.8) |  | 20/27 (74.1) | 10/19 (52.6) | 16/30 (53.3) | 5/12 (41.7) | 2/3 (66.7) |  |
| 11 | 18/30 (60.0) | 12/19 (63.2) | 11/19 (57.9) | 15/24 (62.5) | 31/36 (86.1) | 12/19 (63.2) | 19/27 (70.4) | 9/13 (69.2) |
| 12 | 18/28 (62.1) |  | 4/10 (40.0) | 18/28 (64.3) | 29/30 (96.7) | 11/14 (78.6) | 11/22 (50.0) | 14/24 (58.3) |
| 13 | 29/42 (69.0) | 6/12 (50.0) | 23/34 (67.6) | 17/19 (58.6) | 24/36 (66.7) | 16/24 (66.7) | 11/22 (50.0) | 12/15 (80.0) |
| 14 | 11/15 (73.3) |  | 11/15 (73.3) | 12/21 (57.1) | – | 10/24 (41.7) | 10/16 (62.5) | 8/16 (50.0) |
| 15 | 13/22 (59.1) | 11/15 (73.3) | 16/25 (64.0) | 7/12 (58.3) | – | 10/13 (76.9) | – | – |
| 16 | 17/28 (60.7) |  | 8/14 (57.1) |  | – |  | 8/12 (66.7) | – |
| 17–18 |  | – | 8/14 (57.1) | – | – | 11/15 (73.3) | 8/14 (57.1) | – |
| All | 138/232 (59.5) | 36/61 (59.0) | 134/210 (63.8) | 105/170 (61.8) | 125/170 (73.5) | 88/150 (58.7) | 82/138 (59.4) | 59/99 (59.6) |
| Total | 174/293 (59.4) | | 239/380 (62.9) | | 213/320 (66.6) | | 141/237 (59.5) | |
