# Supplementary material for: Is “Football for All” Safe for All? Cross-Sectional Study of Disparities as Determinants of 1-Year Injury Prevalence in Youth Football Programs
Source: PLoS One. 2012 Aug 22;7(8):e43795. doi: 10.1371/journal.pone.0043795 (PMC3425492; doi:10.1371/journal.pone.0043795)
Supplement: Table S2 — BMI values for the sample and a Swedish reference population. (DOC) [file pone.0043795.s002.doc]

# Supporting information Table S2.

Age- and gender-Specific BMI (Mean and Standard Deviation (SD)) for the sample and a Swedish reference population reported by Karlberg et al 2001 [37].

|  | Girls | | | | Boys | | | |
| --- | --- | --- | --- | --- | --- | --- | --- | --- |
|  | Sample | | Karlberg 2001 | | Sample | | Karlberg 2001 | |
| Age | Mean | SD | Mean | SD | Mean | SD | Mean | SD |
| 8 | 16.0 | 2.0 | 15.7 | 2.0 | 15.8 | 1.6 | 15.8 | 1.5 |
| 9 | 16.6 | 1.5 | 16.1 | 2.2 | 17.2 | 2.5 | 16.1 | 1.7 |
| 10 | 16.8 | 2.5 | 16.6 | 2.5 | 17.1 | 2.0 | 16.5 | 1.9 |
| 11 | 17.8 | 2.4 | 17.1 | 2.8 | 17.5 | 2.0 | 17.0 | 2.2 |
| 12 | 17.6 | 1.9 | 17.8 | 2.9 | 18.5 | 2.2 | 17.6 | 2.5 |
| 13 | 19.1 | 2.5 | 18.4 | 3.0 | 18.7 | 1.8 | 18.2 | 2.7 |
| 14 | 19.6 | 1.8 | 19.1 | 3.1 | 19.7 | 2.6 | 18.9 | 2.9 |
| 15 | 20.4 | 3.4 | 19.8 | 3.0 | 20.3 | 1.6 | 19.6 | 2.9 |
| 16 | 21.6 | 2.3 | 20.4 | 2.9 | 21.5 | 1.6 | 20.3 | 2.9 |
| 17–18*^a^* | 21.8 | 1.5 | 21.0 | 2.8 | 21.8 | 1.9 | 21.1 | 2.8 |

*^a^* Values from Karlberg 2001 are given for 17 years of age.
