# Supplementary material for: Is “Football for All” Safe for All? Cross-Sectional Study of Disparities as Determinants of 1-Year Injury Prevalence in Youth Football Programs
Source: PLoS One. 2012 Aug 22;7(8):e43795. doi: 10.1371/journal.pone.0043795 (PMC3425492; doi:10.1371/journal.pone.0043795)
Supplement: Text S1 — Details of research program. (DOC) [file pone.0043795.s003.doc]

# Supporting information Text S1.

This research is part of the *Play Smart – Be Safe* program for study of safety in youth soccer. The questionnaire was developed to provide information about the safety and security of youth football players in connection with training and matches. Some of these aspects are illustrated in this article and other topics will be presented in future articles. Our model is based on the epidemiologic model of Meeuwisse (1994) in combination with the socioenvironmental framework described by Timpka et al (2006, p. 738) and includes a multifactorial approach that helps to visualize the internal and external modifying conditions and mediating mechanisms, as well as the inciting event and the dispositions of the injured sports person (post-event phase). Items explored whether football players used protective equipment and were encouraged to drink fluids regularly, warm up, cool down at training and in competition, and awareness of injury prevention strategies. These questions have previously been tested for inter-rater reliability, test-retest reliability and content and face validity (Donaldson et al., 2003). Moreover, questions previously validated and used in the research project, School - Sports - Health (SIH project) (<http://www.gih.se/FORSKNING/>), exploring children's and adolescents' self-reported health (illness, injury, abuse event), gender, age, body composition, socioeconomic status, physical capacity and the number/duration of training/match sessions, were included. Finally, the questionnaire was tested a second time for its content and face validity by addressing it to youth football players; minor language and layout changes were here made.

Donaldson A, Hill T, Finch C, Forero R (2003) The development of a tool to audit the safety policies and practices of community sports clubs. *J Sci Med Sport* 6(2):226–230.

School - Sports - Health (SIH project) website (in Swedish). Available: <http://www.gih.se/FORSKNING/Forskningsgrupper/Pedagogik/Pagaende-projekt/Skola---Idrott---Halsa/> Accessed: 2011 Nov 1.

Meeuwisse WH (1994) Assessing causation in sport injury: a multifactorial model. *Clin J Sport Med* 4:166–170.

Timpka T, Ekstrand J, Svanström L (2006) From sports injury prevention to safety promotion in sports. *Sports Med* 36(9):733–745.
